# Case Report: Managing Odontoma and Molar-Incisor-Hypomineralization Challenges in Orthodontics

**DOI:** 10.12688/f1000research.154672.1

**Published:** 2024-08-27

**Authors:** Nora Alhazmi, Khalid Albawardi, May Aleraij, Maram A. Alqahtani, Faisal O. Alsharif, Sohaib Shujaat

**Affiliations:** 1 King Abdullah International Medical Research Center, Riyadh, 11481, Saudi Arabia; 2 Department of Orthodontics, King Abdulaziz Medical City, Ministry of the National Guard Health Affairs, Riyadh, Riyadh Province, 11426, Saudi Arabia; 3 Preventive Dental Science Department, College of Dentistry, King Saud bin Abdulaziz University for Health Sciences, Riyadh, 14611, Saudi Arabia; 4 Department of Restorative Dentistry, King Abdulaziz Medical City, Ministry of the National Guard Health Affairs, Riyadh, 11426, Saudi Arabia; 5 Department of Oral and Maxillofacial Surgery, King Abdulaziz Medical City, Ministry of the National Guard Health Affairs, Riyadh, 11426, Saudi Arabia; 6 OMFS IMPATH Research Group, Department of Imaging and Pathology, Faculty of Medicine, KU Leuven & Department of Oral and Maxillofacial Surgery, University Hospitals Leuven, Ministry of the National Guard Health Affairs, Leuven, 3000, Belgium; 7 Department of Maxillofacial Surgery and Diagnostic Sciences, College of Dentistry, King Saud bin Abdulaziz University for Health Sciences, Riyadh, 14611, Saudi Arabia

**Keywords:** orthodontic; hypomineralization; odontoma; impaction; case report

## Abstract

This report presents the successful orthodontic management of a case of odontoma associated with impaction of the mandibular right permanent canine and Molar-incisor-hypomineralization (MIH). Although case studies have reported the management of odontomas and MIH as separate entities, there is a lack of evidence regarding the comprehensive management of patients presenting with a combination of odontoma, dental impaction, and MIH. A 15-year, 7-month-old female patient complained of the appearance of her smile and the delayed eruption of her mandibular right permanent canine. She was diagnosed with Angle’s Class I molar relationship and Class II canine relationship with the maxillary permanent right lateral incisor in a lingual crossbite. Furthermore, she had mandibular right permanent canine impaction, retained mandibular right primary canine, and MIH in the permanent anterior teeth and first molars. The management involved the removal of the odontoma to allow for orthodontic traction of the lower permanent right canine. After that, the esthetic appearance of the teeth was improved through restorative treatment.

## 1. Introduction

Odontoma, a common odontogenic tumor, has a prevalence ranging from 21% to 67% among all oral tumors. ^1^^,^^2^ These tumors can be classified into two types based on their morphological features: compound and complex. ^3^ In compound odontomas, enamel and dentin are deposited in a way that the resulting structure resembles normal teeth. On the other hand, complex odontomas exhibit irregular masses of dental tissues. Compound odontomas are more frequent than complex ones. Approximately half of these abnormalities are associated with the impaction of permanent teeth, especially canines. ^4^ The presence of odontoma can disrupt the eruption of permanent teeth, which not only compromises occlusal function and aesthetics but may also adversely affect adjacent teeth by causing root resorption.

Moreover, MIH is a prevalent dental developmental disorder that affects approximately 10% to 20% of children and adolescents. ^5^ MIH originates systemically and involves hypomineralization of one to four permanent first molars, often accompanied by affected incisors. The etiology of MIH remains elusive, but it is likely multifactorial, resulting from various environmental factors acting systemically, including prenatal, perinatal, and childhood medical conditions. ^5^ Clinically, teeth affected by MIH exhibit distinct areas of enamel opacities with alterations in translucency, showing a wide variation in color, size, and shape. It can significantly impact a patient’s oral health-related quality of life, causing functional difficulties and aesthetic concerns. ^6^ The best long-term solution involves a combination of minimally invasive treatment options, such as external bleaching, microabrasion, and resin infiltration. However, these treatments may interfere with orthodontic procedures, especially during the adhesion of fixed appliances. ^7^ Additionally, hypomineralized enamel may not withstand forces during active orthodontic treatment or appliance debonding. ^8^

Numerous case studies have documented dental and orthodontic management of odontomas and molar-incisor-hypomineralization (MIH) as separate entities. However, there is a lack of evidence regarding the comprehensive management of patients presenting with a combination of odontoma, dental impaction, and MIH. From a clinical perspective, it is crucial for practitioners to be familiar with the comprehensive management of such cases to enhance treatment outcomes and current standards of care. Therefore, the aim of this case report was to present a multidisciplinary approach for managing a patient with a compound odontoma associated with an impacted mandibular canine and MIH.

## 2. Etiology and diagnosis

This case report was conducted in compliance with the World Medical Association Declaration of Helsinki on medical research. Ethical approval was obtained from the Ethical Review Board of the University Hospital (NRR24/061/5). A written informed consent was obtained from the patient to publish the current case report and accompanying images. In addition, the Consensus-based Clinical Case Reporting Guideline Development (CARE) guidelines checklist was followed. ^9^

A 15-year and 7-month-old female patient presented to the orthodontic clinic at University Hospital. The main concern of the patient was the appearance of her smile, and she expressed worry about the delayed eruption of her mandibular right permanent canine. The patient had no history of trauma. Furthermore, the patient had iron deficiency anemia and vitamin D deficiency, and she irregularly took iron and vitamin D supplements. Patient also had a history of antibiotics administration for fever at the age of 2 years. During an investigation into teeth discoloration, the patient’s mother reported experiencing stress during pregnancy and had a history of urinary tract infection, which was treated with antibiotics. Additionally, the mother reported prolonged labor during childbirth.

### 2.1 Clinical examination

Upon extraoral examination, a convex profile with competent lips was observed in the lateral view. In the frontal view of the non-smiling face, a deviated nose and symmetrical features were evident. When smiling, the frontal view revealed an average smile line with a 90% incisal display, a non-consonant smile arc, and a wide buccal corridor. Notably, there was a 1 mm deviation of the maxillary dental midline to the right relative to the facial midline, with the lower midline coinciding relative to the facial midline.

**Figure 1.**
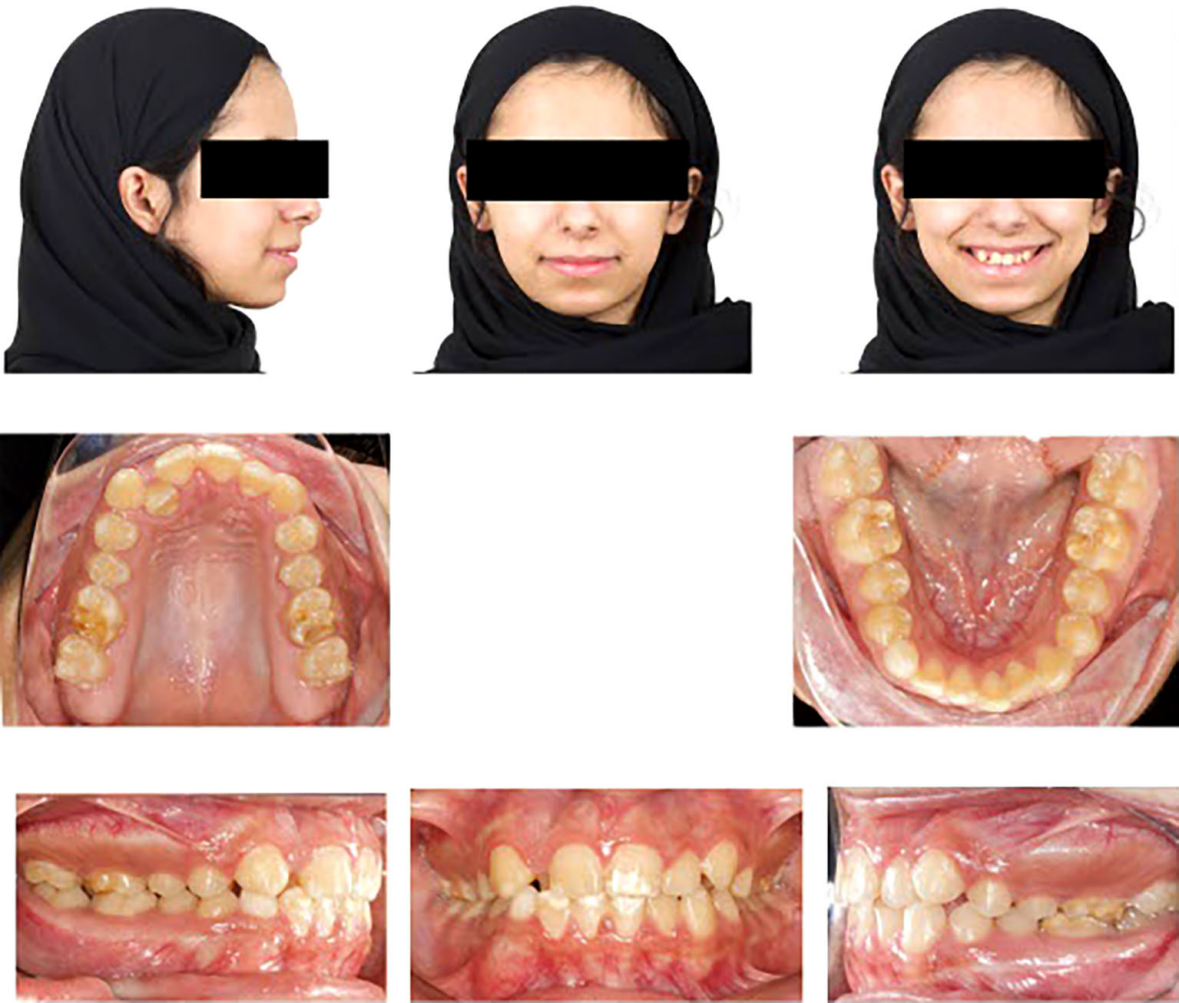
Extra-oral and intra-oral baseline presentation.

During intraoral examination, no carious lesions were found. However, mild plaque-induced gingivitis was present around all anterior permanent teeth. The patient exhibited an Angle’s Class I relationship. ^10^ Canine relationships were classified as Class II. Specifically, the upper right permanent lateral incisor was lingually displaced and in a lingual crossbite. Retained lower right primary canine was the result of the odontoma that caused impaction of the lower right permanent canine. Additionally, there was bulging of the alveolar vestibule below the lower right primary canine. The overjet measured 2 mm, and the overbite was 17%. White, yellow, and brown stains were visible on the buccal and occlusal surfaces of the upper and lower permanent first molars. Facial surfaces of the upper right canine, upper central incisors, lower central incisor, and lower right lateral incisor exhibited white or creamy stains. Furthermore, the tooth size-arch length discrepancy was -4 mm in the maxillary arch and -2 mm in the mandibular arch, indicating mild upper and lower crowding ( Figure 1). ^11^

### 2.2 Radiographic examination

Panoramic radiograph was acquired, which showed a radiopaque mass, consisting of multiple small, calcified tooth-like structures, surrounded an oval radiolucent lesion around the crown of the unerupted lower right permanent canine. No root resorption was observed, and no other anomalies or pathologies were noted. Cone-beam computed tomography was performed which confirmed the radiological diagnosis of compound odontoma with impaction of the lower right permanent canine.

Lateral cephalometric radiography indicated cervical vertebral maturation stage V, suggesting that the peak of mandibular growth occurred no earlier than 2 years before this stage. ^12^ The anteroposterior position of the maxillary and mandibular jawbones showed an SNA of 81°, an SNB of 77°, and an ANB of 4°, indicating skeletal class II due to the retrognathic mandible. The Frankfort-mandibular plane angle (FMA) measured 24°, with a normodivergent mandibular plane angle.

**Figure 2.**
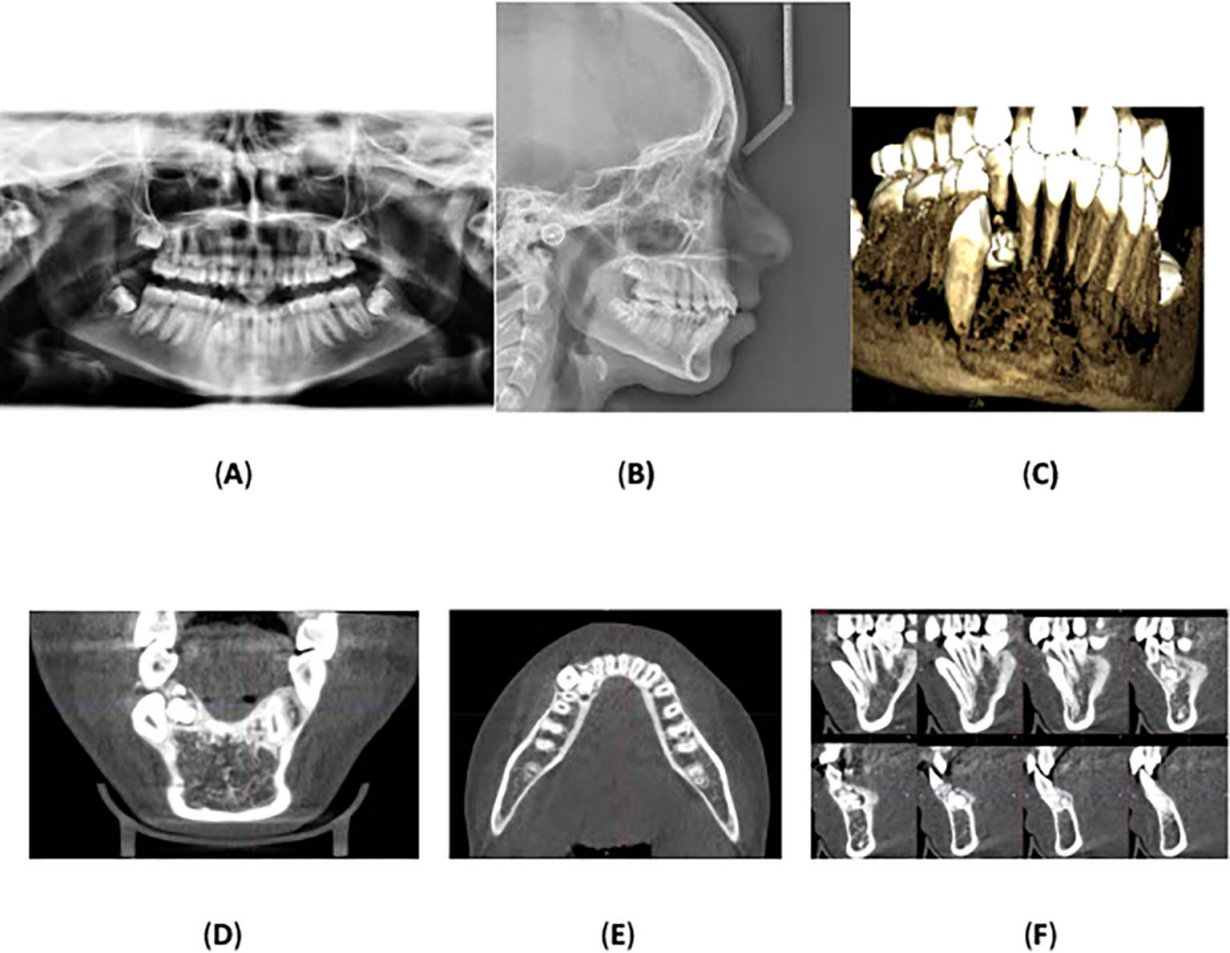
Radiographs: (A) orthopantomography, (B) lateral cephalometric, and (C) cone-beam computed tomography showing the impaction of the lower right permanent canine with the presence of a compound odontoma. (D) and (E) Coronal and axial view showing the location of the odontoma on the lingual aspect of the alveolar bone and the impacted lower permanent right canine is located labially towards the vestibulum. (F) A multi surface axial view showing that the odontoma occupies a large area between the lingual and labial surface of the alveolar bone.

Regarding the upper anterior relation to the anterior cranial base, the SN-U1 angle was 104° (within the normal range), and the mandibular plane angle (IMPA) and Frankfort-mandibular incisor angle (FMIA) were 97° and 58°, respectively, indicating lingual inclination of the mandibular anterior teeth. The upper and lower lip positions relative to Ricketts’ esthetic line (E-line) were -2 mm and 2 mm, respectively, indicating a normal lip position. ^13^^,^^14^ Figure 2A-F displays the radiographs, and Table 1 summarizes the cephalometric analysis.

**Table 1.** Pre-treatment and post-treatment cephalometric analysis.

Landmarks	Pre-treatment (15 year, 7-month-old)	Post-treatment (17 year, 8-month-old)
SNA (°)	81.00	81.00
SNB (°)	77.00	77.00
ANB (°)	04.00	04.00
FMA (°)	25.00	26.00
SN-U1 (°)	104.00	104.00
IMPA (°)	94.00	98.00
FMIA (°)	61.00	60.00
U1-NA (°)	22.00	20.00
L1-NB (°)	25.00	29.00
U1/L1 (°)	130.00	126.00
Upper lip to E-line (mm)	-01.00	-02.00
Lower lip to E-line (mm)	02.00	01.00

### 2.3 Diagnostic assessment

**Figure 3.**
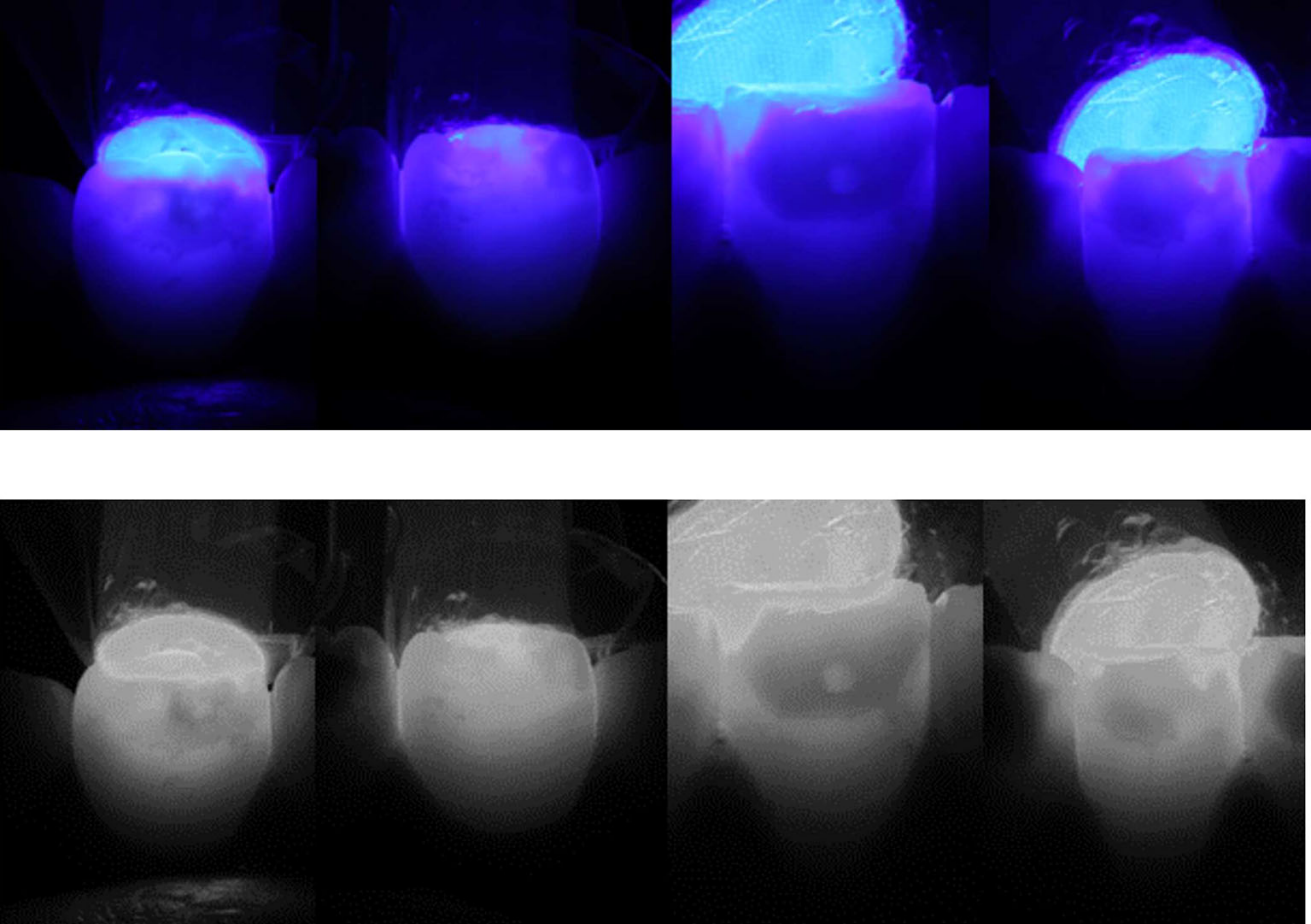
Transillumination assessment positioning the tip of the light-curing unit at the palatal/lingual surfaces. Dark and intense color indicates deep stains.

The clinical appearance, combined with the radiographic findings, showed a diagnosis of compound odontoma associated with impacted mandibular right permanent canine. Transillumination assessment of the stains was performed by positioning the tip of a light-emitting diode (LED) light-curing unit at the palatal/lingual surfaces of teeth. Dark and intense color was observed which indicated towards deep stains ( Figure 3). Considering both clinical and medical history, MIH was also diagnosed. According to the MIH-Treatment Need Index (MIH-TNI) criteria, the case was classified as MIH without hypersensitivity and without defect extension (MIH TNI 1). ^5^

## 3. Surgical intervention

The patient underwent surgical extraction of the lower right primary canine and excision of the odontoma. The surgical incision extended from the lower right second premolar to the lower right permanent central incisor. A distal releasing incision was made near the lower right second premolar, followed by subperiosteal dissection on the lingual side where the bulge was located. The odontoma was then removed intact. A subperiosteal flap exposed the lower right permanent canine, and buttons were placed on its lingual surface for traction. The biopsy sample was sent to the Oral Pathology Department, confirming a diagnosis of compound odontoma.

## 4. Orthodontic intervention

### 4.1 Orthodontic treatment

The bonding of all upper and lower permanent teeth (except the lower right permanent canine) was carried out using a pre-adjusted edgewise appliance (0.018” × 0.025”) with the MBT prescription system (3M Unitek, Monrovia, California, USA) and 3M ^TM^ Transbond XT Light Cure Adhesive (3M company, St. Paul, Minnesota, USA). Additionally, the bracket of the upper right permanent lateral incisor was reversed to torque the root forward. Orthodontic bands were applied instead of molar tubes on all permanent first molars to enhance retention due to compromised enamel mineralization. Notably, the bracket bonding strength was suitable for both the upper and lower anterior teeth, and the patient did not encounter any instances of bracket bonding failure.

Initially, the upper and lower dentitions were leveled and aligned using 0.014-inch and 0.016-inch nickel-titanium (NiTi) archwires. Subsequently, 0.016-inch stainless steel (SS) archwires were prescribed. After 6 months of treatment, a follow-up panoramic radiograph was taken to assess root parallelism. Some brackets were repositioned, and the archwires were downgraded to 0.016-inch NiTi. Later, the wires were progressed to 0.016-inch by 0.022-inch SS. An open-coil spring was then used to create space for the upper right permanent incisors and shift the upper midline toward the left side. Additionally, another open-coil spring was placed to create space for the lower right permanent canine. Traction was applied to the lower right permanent canine using an elastic chain with an average force of 60 grams. After 3 months, the lower right permanent canine began to erupt. Lingual root torque was applied to this tooth during the finishing stage. Finally, a 0.017-inch by 0.025-inch SS wire, along with class II elastics (60 grams), was used to achieve a class I canine relationship. Retention included a lower fixed retainer with an overlay Hawley retainer, while the upper arch was retained using an upper Hawley retainer. The total treatment duration was 25 months.

### 4.2 Post-orthodontic assessment

**Figure 4.**
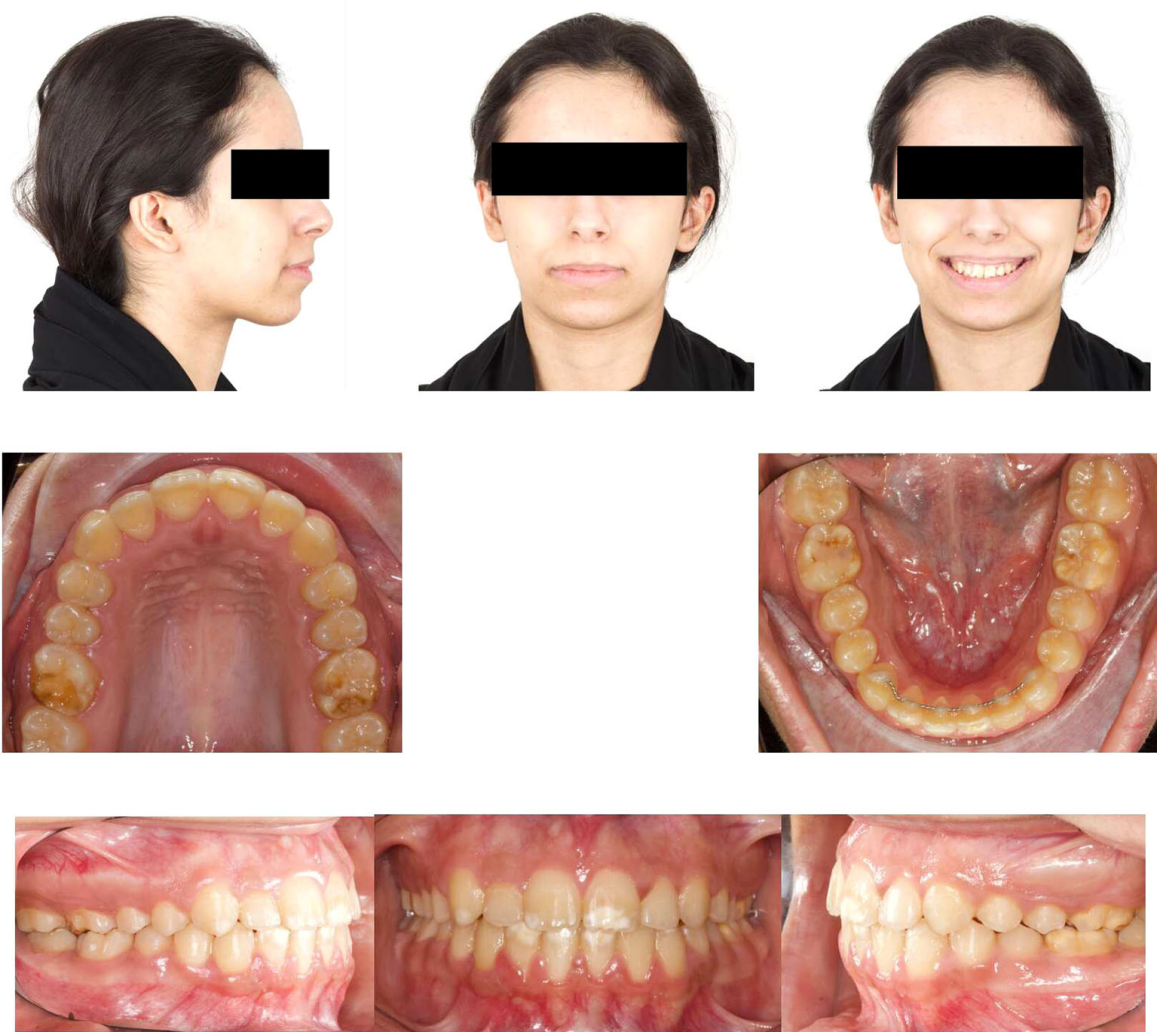
Final extra-oral and intra-oral photographs.

Post-orthodontic follow-up was conducted at the time-point of 26 months. Upon examining the frontal view of the face, the maxillary dental midline aligned precisely with the lip philtrum and facial midline. The smile, as seen from the frontal view, displayed a harmonious smile arc with full visibility of the upper incisors. Intraoral examination revealed an Angle’s class I relationship, with appropriate overjet and overbite for the canines ( Figure 4). Panoramic radiography confirmed proper root parallelism without any periodontal abnormalities. Additionally, both upper and lower third molars were in the developmental stage.

The cephalometric evaluation showed no significant change in both the SNA and SNB angles. The FMA angle increased slightly by 1°, indicating a minor clockwise rotation of the mandible. Dental analysis showed that the SN-U1 angle remained unchanged, but U1-NA decreased by 2°, suggesting forward displacement of the Nasion landmark. For the mandibular anterior teeth, the L1-NB angle increased by 4°, IMPA increased by 4°, and FMIA decreased by 1°, indicating labial tipping of the lower central incisors. The inter-incisal angle decreased by 4°, reflecting proclination of both upper and lower anterior teeth.

**Figure 5.**
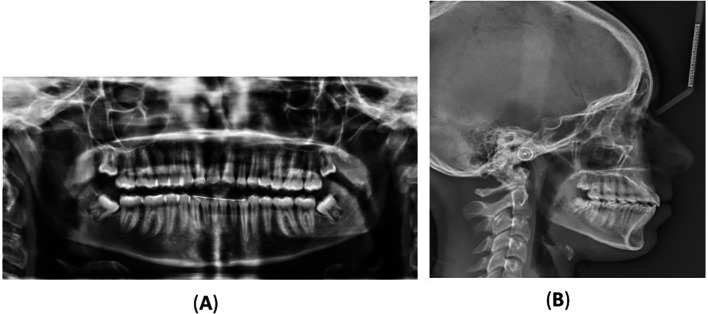
Final radiographs: (A) orthopantomography and (B) lateral cephalometric.

**Figure 6.**
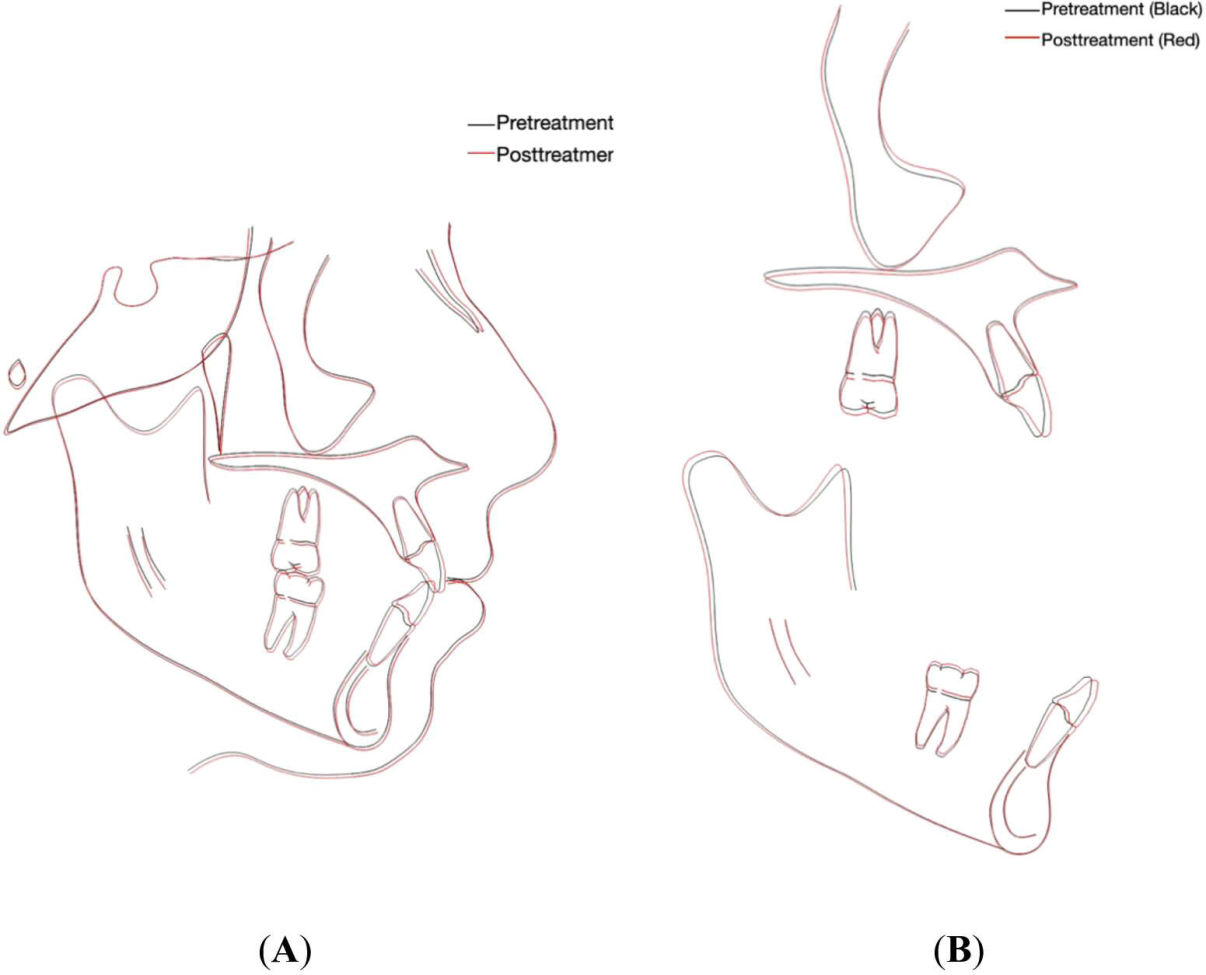
Cephalometric superimposition. (A) Overall superimposition: the black tracing indicates pretreatment (15 years, 7 months) and the red tracing indicates posttreatment (17 years, 8 months). (B) Maxillary and mandibular regional superimposition; the black tracing indicates pretreatment (15 years, 7 months) and the red tracing indicates posttreatment (17 years, 8 months).

Soft tissue analysis revealed slight retrusion of the upper and lower lips relative to the E line ( Figure 5A, Figure 5B and Table 1). A cephalometric superimposition was performed using the Dolphin imaging software (Dolphin version 11.59, Dolphin Imaging and Management Solutions, Chatsworth, CA, USA) available at ( https://www.dolphinimaging.com), which showed slight extrusion of the permanent first molars and proclination of upper and lower incisors ( Figure 6A and Figure 6B). Following completion of orthodontic treatment, the patient was referred to a restorative dentist to enhance the aesthetics of the hypomineralized anterior teeth.

## 5. Restorative interventions

### 5.1 Management of MIH

Preventive strategies included educating both parents and patient about dental hygiene and caries prevention procedures at home, such as the use of fluoridated toothpaste. In-office measures included application of Fisseal Light Curing Pit and Fissure Sealant with Fluorides (PROMEDICA Dental Material GmbH, Germany) and professional fluoride in the form of gel to minimize dental caries and sensitivity. In addition, NovaMin ^®^ (GlaxoSmithKline Consumer Healthcare, Weybridge, Surrey, UK) dentifrice was prescribed to enhance dental remineralization and desensitization. ^5^

### 5.2 Restoration of anterior teeth

5.2.1 Bleaching

**Figure 7.**
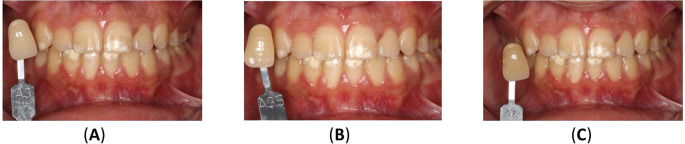
Initial shades of the teeth using the VITAPAN® Classical visual scale. (A) The upper incisors were presented in shade A3, and the canines in shade A3.5. (B) The lower teeth were presented in shade A3.5 for incisors, and (C) in shade A4 for canines.

In light of the patient’s age, a minimally invasive approach was suggested to enhance the aesthetic condition of the teeth rather than traditional restorative treatment. Initially, dental prophylaxis using a non-drying, splatter-free Qartz prophy paste containing 1.23% fluoride and 0.1 molar phosphate (Pearson ^TM^ Dental, Sylmar, CA, USA) mixed with water (2:1 ratio) and supragingival scraping with periodontal curettes were used to stabilize the oral environment. In the subsequent session, the initial tooth shade was visually assessed using validated method the VITA classical A1-D4 ^®^ shade guide. Required license has been obtained for the use of this tool (VitaZähnfabrik, Bad Säckingen, Germany). ^15^ The upper incisors ranged from shades A3 to A3.5, while the canines were shade A3.5. The lower incisors were shade A3.5, and the canines were A4 ( Figure 7A-C).

**Figure 8.**
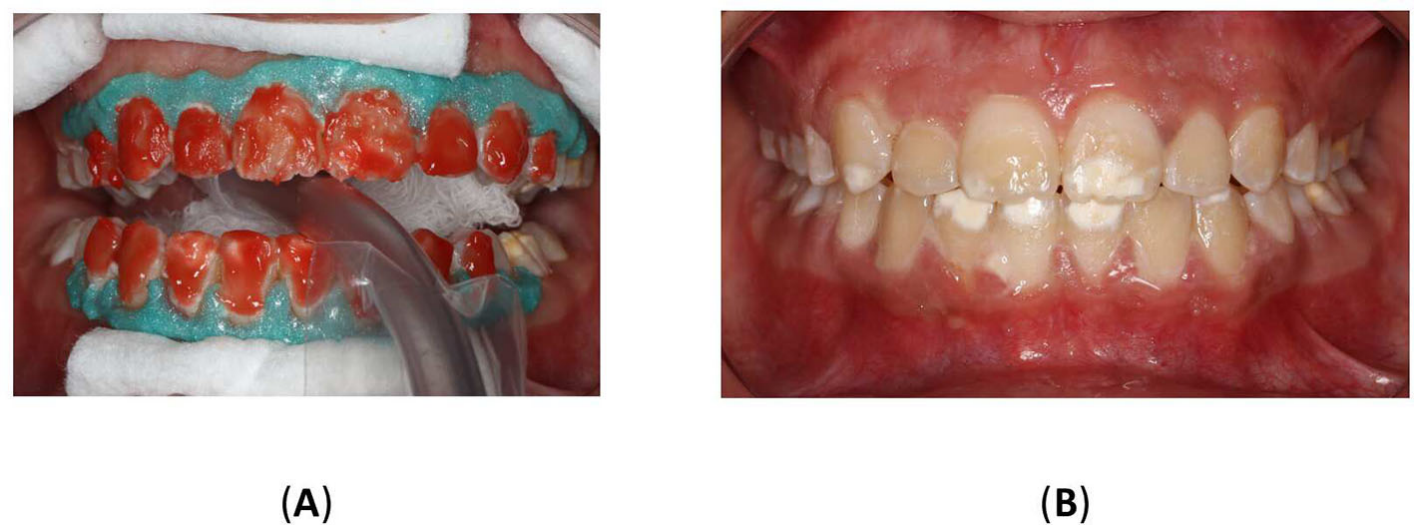
(A) Dental Bleaching Opalescence Boost 40% (Ultradent Products); (B) NovaBright™ 5% sodium fluoride varnish was applied and lasted for 10 min.

A digital impression was obtained using 3Shape CAD/CAM TRIOS ^®^ 4 and bleaching trays were created using Rubber Transparent (3A Medes Easy Vac Gaskets, India). Before using the trays, their proper adaptation to the teeth and gingival tissues was assessed. At the beginning of the bleaching session, a gingival barrier (OpalDam ^®^, Ultradent Products, Inc., South Jordan, Utah, USA) was applied and light-cured for 20 seconds on the gingival contour of all teeth to protect against mucosal irritation. For the home bleaching technique, Opalescence ^®^ Boost 40% (Ultradent Products, Inc., South Jordan, Utah, USA) was used, a hydrogen peroxide-based agent prepared according to the manufacturer’s instructions. Two 20-minute applications of the bleaching agent were performed. After each session, the teeth were thoroughly rinsed with water. Subsequently, neutral colorless NovaBright™ 5% sodium fluoride varnish (Nanova Biomaterials, Inc., Columbia, Missouri, USA) was applied and allowed to dry for 10 minutes ( Figure 8A-B).

To achieve more satisfactory aesthetic results, a second home bleaching treatment was performed using carbamide peroxide gel Opalescence™ PF 35% (Ultradent Products, Inc., South Jordan, UTAH, USA). The treatment involved daily application for 30 to 60 minutes over two weeks, followed by the use of sodium fluoride varnish. In the first week, the bleaching material was applied daily, and in the second week, it was used every day. At the end of each week the patient’s teeth and gingival tissues were evaluated. The patient received tooth whitening instructions, including tips to avoid coffee, soft drinks, and citrus fruit juices.

**Figure. 9.**
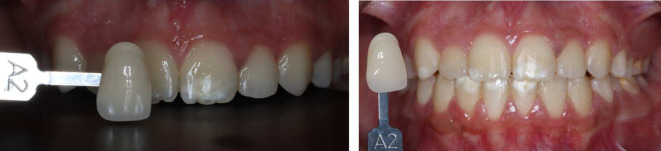
Final color of teeth after home bleaching using the VITAPAN ^®^ Classical visual scale.

After both bleaching procedures were finished, a 2-week waiting period was observed before the final shade evaluation during the patient’s recall visit. The patient’s teeth appeared brighter, with noticeable improvement in previously discolored areas. Figure 9 illustrates the results of home bleaching, showing the upper incisors, upper canines, lower incisors, and lower canines in shade A2.

5.2.2 Resin infiltration

**Figure 10.**
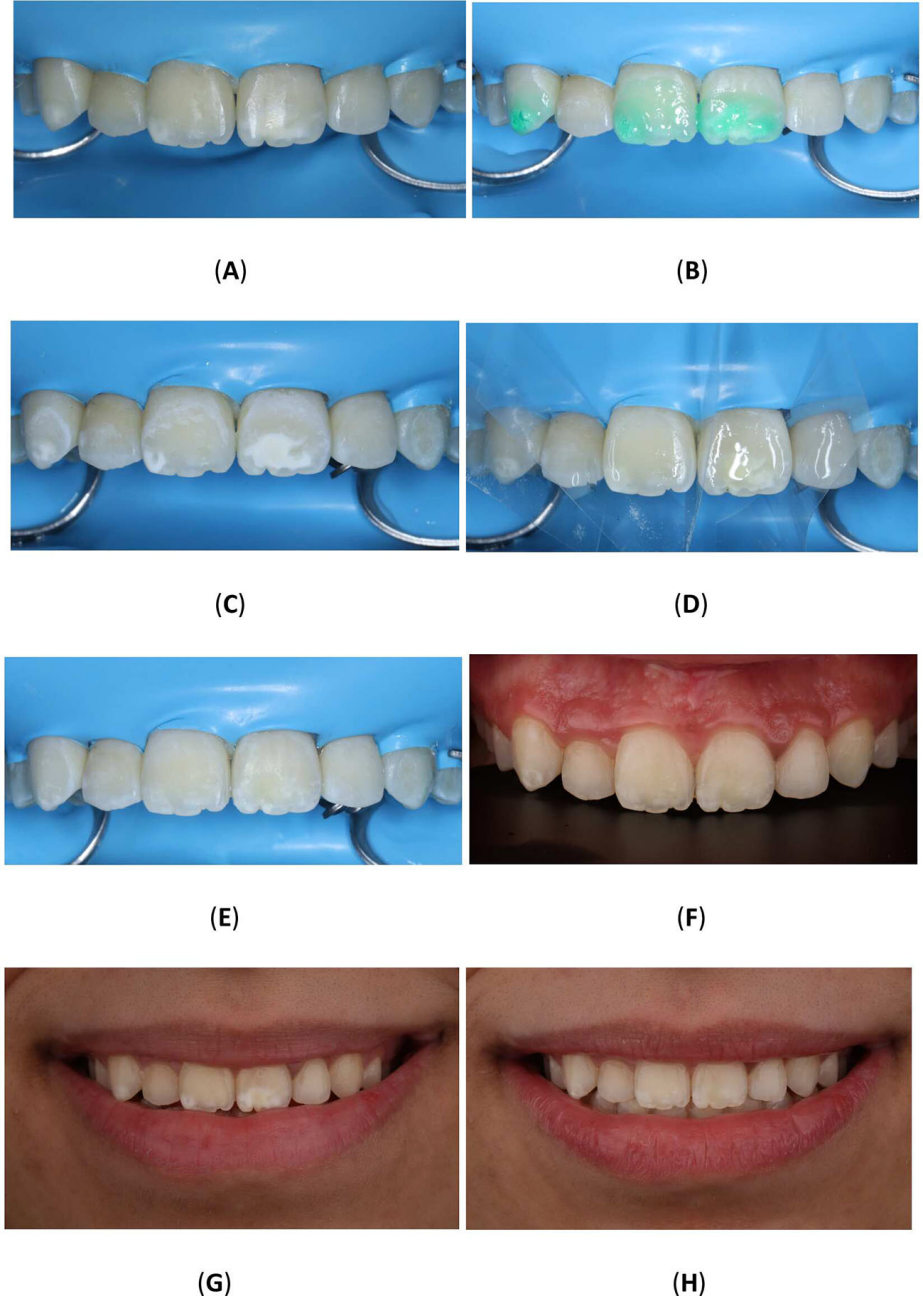
(A) Steps in Icon application: Isolation with rubber dam; (B) Acid application with etch syringe; (C) Ethanol application; (D) Application of resin; (E) Clinic photography post-treatment; (F) Upper teeth with contrast; (G) Frontal smile before Icon application; (H) Frontal smile after Icon application.

To improve the translucency and esthetics of teeth, resin infiltration was planned as next step after bleaching. The Icon ^®^ Vestibular (DMG, Hamburg, Germany) was performed according to the manufacturer’s instructions. The affected teeth were isolated using a rubber dam (Sanctuary ^®^ Powder-free Latex Silk Blue Dental Dam, Malaysia) and a clamp (HuFriedyGroup, STERIS, USA). A 15% hydrochloric acid was applied to the lesion using the 0.45 ml (Icon Etch, DMG, Hamburg, Germany). After allowing it to sit for 2 minutes, the tooth was rinsed with water for 30 seconds and dried using an air spray. After the third etching round, a 0.45 ml ethanol (Icon Dry, DMG, Hamburg, Germany) was applied to remove the water retained in the microporosities of the enamel, letting it last for 30 seconds. Subsequently, infiltrating resin was applied to the lesion using the 0.45 ml (Icon-Infiltrant, DMG, Hamburg, Germany). The resin infiltrant was massaged onto the lesion surface in circular movements for 3 minutes, ensuring thorough penetration. The excess resin was removed with air-spray and dental floss. Finally, the resin infiltrant was polymerized for 40 seconds using a light-cure device (Planmeca Lumion™ Plus polymerization light, Planmeca OY, Finland). The treatment protocol involved three rounds of hydrochloric acid application (2 minutes each) followed by a 10-minute infiltration period. An additional minute of treatment was administered after the initial infiltrating procedure. Upon completion, the rubber dam and clamps were removed, and surface finishing was performed using fine-grained polishing burs and the dental composite polishing diamond system RA Disc 14mm Wheel (Rubber Wheel, AZDENT, China) ( Figure 10A-H).

5.2.3 *Restoration of posterior teeth*

An indirect onlay restoration was performed on the upper right first molar due to cusp involvement. During the onlay preparation, efforts were made to completely remove the hypomineralized enamel and place the final preparation margins on sound, unaffected enamel. Approximately 1.5 mm reduction was done for the functional cusps (distolingual), and 1 mm reduction for the nonfunctional cusps (distobuccal). A butt joint margin was prepared using a long round end taper (856L) bur. A digital impression was obtained using the 3Shape CAD/CAM TRIOS 4 system. The temporary restoration was fabricated using the Ivoclar Vivadent Systemp. In the laboratory procedure, two sets of casts were printed using JamgHe 10K Standard Plus Resin for an LCD DLP SLA 405nm 3D Printer (High Transparent, 1000 g). The ceramic onlay was created using Ivoclar Vivadent AGFL-9494 Schaan/Liechtenstein in shade A2.

During the try-in visit, the onlay was placed, followed by a bitewing radiograph. Subsequently, the onlay was etched with 9.5% hydrofluoric acid for 20 seconds, and a silane coupling agent (Deutschland GmbH Germany 3M ESPE) was applied for 1 minute. The temporary restoration was removed, and the molar was cleaned and etched using 37% phosphoric acid (Scotchbond ^TM^ Multi-Purpose Etchant, 3M ESPE) for 15 seconds, followed by rinsing with water for 15 seconds and drying with high-volume suction. The bonding agent (Adper ^TM^ Single Bond Plus, 3M ESPE) was actively applied, and dual-cure resin cement (RelyX ^TM^ Unicem 2 Clicker™100, 3M ESPE) was used for final cementation. After cementation, occlusion was assessed, and the onlay was finished and polished.

## 6. Patient follow-up

**Figure 11.**
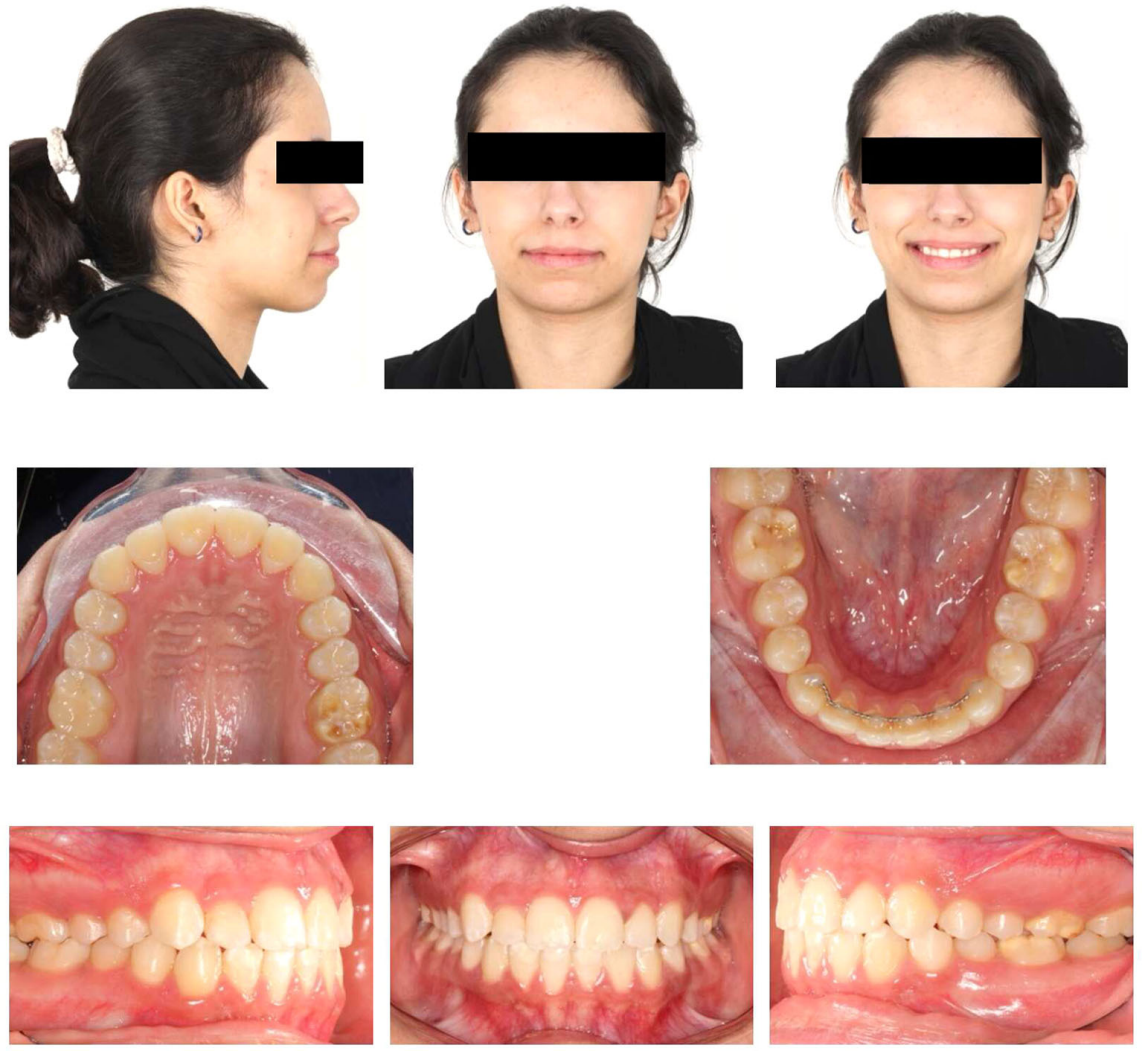
Follow-up extra-oral and intra-oral photos.

The patient was followed up three months later after orthodontic debonding and restorative treatment ( Figure 11). Oral hygiene was reinforced, and retainers were checked. Additionally, the patient exhibited no temporomandibular joint symptoms, and gingiva remained healthy. The patient was satisfied with the treatment outcomes and experienced improvements in esthetics and mastication. Her self-esteem increased significantly. Moreover, she was informed about the potential need for future extraction of all third molars.

## 7. Discussion

The present case report describes a comprehensive management strategy for a patient with compound odontoma associated with impacted canine and MIH. The management involved the removal of odontoma to allow for orthodontic traction of the lower permanent right canine. Thereafter, esthetic appearance of the teeth was improved through restorative treatment.

When considering the presence of odontomas associated with impacted teeth, it is important to highlight that the decision to remove it and extrude the impacted tooth varies based on patient-specificity. The failure rate for traction of the mandibular canines has been documented to be 17%. ^16^ However, successful traction was possible in the present case. At the same instance, it is important to highlight that alternative treatment options should be opted where traction of the mandibular canine could be challenging owing to the deep location of impaction, possibility of ankylosis and presence of critical sized defect following odontoma removal. If ankylosis existed with deep impaction an alternative treatment option would be to extract the lower right permanent canine, excise the odontoma, mesialize the lower right posterior segment, and substitute the lower right first premolar for the lower right permanent canine. In addition, bone grafting should be opted for cases with large defects following odontoma removal, where traction of canine is planned. In the present case no grafting was performed owing to the small dimensions of the odontoma. However, future radiological studies are recommended to assess the impact of grafting and size of defect on the orthodontic traction of impacted teeth following odontoma removal.

In relation to MIH, it is a clinically challenging condition due to the compromised bonding of fixed orthodontic appliances. A failure of bracket system adherence to the defective enamel structure occurs, and the bracket may not be able to withstand active orthodontic forces. However, the presented patient did not experience bracket bonding failure or compromised orthodontic movement, as the hypocalcification was present on the incisal edge of the anterior teeth away from the bracket position. In cases with extended hypocalcification inhibiting the application of brackets, it has been proposed to either apply glass ionomer cement-based adhesives or perform microabrasion on hypomineralized enamel before etching. ^17^ In addition, a study suggested that enamel deproteinization with 5% sodium hypochlorite prior to the bonding procedure might improve the bonding performance of resin adhesives to the hypomineralized enamel. ^18^

Debonding is another challenging factor in cases of MIH. Removal of the fixed orthodontic appliance could create stress on the defective enamel structure and lead to loss of the affected enamel with microfractures. ^8^ In this case, a low-speed white stone bur was used to reduce damage to the enamel structure. A recent study also suggested the use of a laser or thermal method to reduce enamel fractures. ^19^ In addition, hypersensitivity among patients with MIH has been reported owing to increased porosity and high protein concentrations. ^20^ One potential cause of hypersensitivity in MIH is the inflammatory response in the pulp due to oral bacteria penetrating through the hypomineralized enamel into the dentinal tubules. Additionally, tooth hypersensitivity related to MIH also seems to be present in disintegrated molars immediately after tooth eruption. The management of such a condition involves preventive and therapeutic approaches, such as using fluoride varnishes and casein-based products. However, the presented case did not report any sensitivity.

The presented patient experienced unpleasant white spot lesions on the anterior teeth, prompting bleaching and resin infiltration. As a result, smile aesthetics improved, and the patient expressed satisfaction with the final outcome. A recent systematic review also advocated for using resin infiltration to conceal enamel fluorosis, while another study highlighted the effectiveness of bleaching as a treatment approach.

The primary strength of the case report lies in its first-time documentation of the comprehensive management of a patient with compound odontoma associated with an impacted tooth and MIH. However, it is important to interpret the findings with caution, as a single case cannot establish a cause-and-effect relationship. To confirm treatment outcomes and establish patient-specific treatment guidelines, further prospective studies with larger sample sizes and long-term follow-up are recommended.

## 8. Conclusions

The case report highlights the successful management of a patient with compound odontoma, an impacted mandibular canine, and MIH. The treatment approach involved surgically removing the odontoma, followed by orthodontic intervention to guide the impacted canine into its proper position. Due to the presence of MIH, the orthodontic intervention was altered to include orthodontic bands instead of molar tubes, for improved retention and a conservative debonding procedure was performed with a low-speed handpiece. Additionally, cosmetic dental treatment was performed using bleaching and resin infiltration for the anterior teeth, and onlay restoration for maxillary right first molar.

## Author contributions

Conceptualization, N.A. and K.A.; Data curation, K. A. and M.A.A.; formal analysis, K.A., M.A., M.A.A., F.O.A., and S.S.; funding acquisition, N. A.; investigation, N.A., K.A., M.A., M.A.A., and F.O.A.; methodology, N.A.; project administration, N. A.; resources, N.A.; software, K.A.; supervision, N. A., M.A., and S.S.; validation, N.A.; visualization, N. A. and M.A.; writing—original draft preparation, N. A.; writing—review and editing, N. A., K. A., M.A., M.A.A., F.O.A., and S.S.

## Consent statement

A written informed consent was obtained from the patient and parents to publish the current case report and accompanying images.

## Data Availability

No data associated with this article.

## References

[1] Hidalgo-Sánchez O Leco-Berrocal MI Martínez-González JM : Metaanalysis of the epidemiology and clinical manifestations of odontomas. *Med. Oral Patol. Oral Cir. Bucal.* 2008;13(11):730–734. 18978716

[2] Tekkesin MS Pehlivan S Olgac V : Clinical and histopathological investigation of odontomas: review of the literature and presentation of 160 cases. *J. Oral Maxillofac. Surg.* 2012;70(6):1358–1361. 21840103 10.1016/j.joms.2011.05.024

[3] Sarica İ Derindağ G Kurtuldu E : A retrospective study: Do all impacted teeth cause pathology? *Niger. J. Clin. Pract.* 2019;22(4):527–533. 10.4103/njcp.njcp_563_18 30975958

[4] Dunfee BL Sakai O Pistey R : Radiologic and pathologic characteristics of benign and malignant lesions of the mandible. *Radiographics.* 2006;26(6):1751–1768. 17102048 10.1148/rg.266055189

[5] Lygidakis N Garot E Somani C : Best clinical practice guidance for clinicians dealing with children presenting with molar-incisor-hypomineralisation (MIH): an updated European Academy of Paediatric Dentistry policy document. *Eur. Arch. Paediatr. Dent.* 2022;23(1):3–21. 10.1007/s40368-021-00668-5 34669177 PMC8926988

[6] Silva MJ Scurrah KJ Craig JM : Etiology of molar incisor hypomineralization–A systematic review. *Community Dent. Oral Epidemiol.* 2016;44(4):342–353. 10.1111/cdoe.12229 27121068

[7] Elhennawy K Schwendicke F : Managing molar-incisor hypomineralization: A systematic review. *J. Dent.* 2016;55:16–24. 10.1016/j.jdent.2016.09.012 27693779

[8] Koletsi D Gerald Bradley T Kavvadia K : Bonding and Debonding Considerations in Orthodontic Patients Presenting Enamel Structural Defects. *Debonding and Fixed Retention in Orthodontics: An Evidence-Based Clinical Guide.* 2023; pp.43–62.

[9] Riley DS Barber MS Kienle GS : CARE guidelines for case reports: explanation and elaboration document. *J. Clin. Epidemiol.* 2017;89:218–235. 10.1016/j.jclinepi.2017.04.026 28529185

[10] Katz MI : Angle classification revisited 2: a modified Angle classification. *Am. J. Orthod. Dentofacial Orthop.* 1992;102(3):277–284. 1510054 10.1016/S0889-5406(05)81064-9

[11] Kirschen RH O’Higgins EA Lee RT : The Royal London Space Planning: an integration of space analysis and treatment planning: Part I: Assessing the space required to meet treatment objectives. *Am. J. Orthod. Dentofacial Orthop.* 2000;118(4):448–455. 11029742 10.1067/mod.2000.109031

[12] Baccetti T Franchi L McNamara JA Jr : An improved version of the cervical vertebral maturation (CVM) method for the assessment of mandibular growth. *Angle Orthod.* 2002;72(4):316–323. 12169031 10.1043/0003-3219(2002)072<0316:AIVOTC>2.0.CO;2

[13] Ng JHH Singh P Wang Z : The reliability of analytical reference lines for determining esthetically pleasing lip position: An assessment of consistency, sensitivity, and specificity. *Am. J. Orthod. Dentofacial Orthop.* 2023 Jul;164(1). 10.1016/j.ajodo.2023.04.011 37227323

[14] Ricketts RM : Planning treatment on the basis of the facial pattern and an estimate of its growth. *Angle Orthod.* 1957;27(1):14–37.

[15] Meireles SS Demarco FF Santos IS : Validation and reliability of Visual Assessment with a shade guide for tooth-color classification. *Oper. Dent.* 2008 Mar 1;33(2):121–126. 10.2341/07-71 18435184

[16] Dalessandri D Parrini S Rubiano R : Impacted and transmigrant mandibular canines incidence, aetiology, and treatment: a systematic review. *Eur. J. Orthod.* 2017;39(2):161–169. 10.1093/ejo/cjw027 27036134

[17] Marure PS Mahamuni A Ambekar AS : Orthodontic bracket bonding challenge for fluorosed teeth. *J. Int. Oral Health.* 2016;8(4):476–480.

[18] Ekambaram M Yiu C : Bonding to hypomineralized enamel–A systematic review. *Int. J. Adhes. Adhes.* 2016;69:27–32.

[19] Gibas-Stanek M Pełka P Pihut M : What is the safest method of orthodontic debonding - a systematic review of the literature. *Folia Med. Cracov.* 2023;63(3):133–156. 10.24425/fmc.2023.147219 38310534

[20] Lyaruu D Bervoets T Bronckers A : Short exposure to high levels of fluoride induces stage-dependent structural changes in ameloblasts and enamel mineralization. *Eur. J. Oral Sci.* 2006;114 Suppl 1:111–115. 16674671 10.1111/j.1600-0722.2006.00346.x

