# Supplementary material for: Case Report: Managing Odontoma and Molar-Incisor-Hypomineralization Challenges in Orthodontics
Source: F1000Res. 2025 Jul 23;13:970. Originally published 2024 Aug 27. [Version 3] doi: 10.12688/f1000research.154672.3 (PMC12188183; doi:10.12688/f1000research.154672.3)
Supplement: Peer review history

# Reviewer report: Reviewer response for version 2

**DOI:** 10.5256/f1000research.178206.r390520

**Published:** 2025-06-24

**Authors:** Afrah A. K. Aldelaimi

**Affiliations:** 1 University Of Anbar, Ramadi, Iraq

*• The manuscript within the scope of the journal. *

- *The manuscript's quality and data presentation are acceptable and important for clinicians, pathologists and even patients.*
* • The manuscript advances our understanding of  odontogenic tumors and cyst  *
- *The title should be rewritten to be more precise and explanatory.*
* • Make the abstract more informative and it should represent the article's substance and be no more than 250 words long.*
* • Include four to six keywords that are relevant to the manuscript but not stated in the title.*
* • Additional paragraphs to introduction about maxillary sinus diseases, cyst and tumors and its managements *
.

Suggested References:

 refer to 1, 2 and 3

  

- Care should be taken to improve resolution and contrast for each figure in the manuscript and arrows to each picture for illustration purposes.
  • Authors should check for writing and typing errors.
  • The statements in discussion are acceptable but few paragraphs about the justification of your findings and comparison with other recent relevant studies.

• Only include current up to date references in the reference list and remove outdated ones.

  Good Luck

Are enough details provided of any physical examination and diagnostic tests, treatment given and outcomes?

No

Is the case presented with sufficient detail to be useful for other practitioners?

Yes

Is sufficient discussion included of the importance of the findings and their relevance to future understanding of disease processes, diagnosis or treatment?

No

Is the background of the case’s history and progression described in sufficient detail?

Yes

Reviewer Expertise:

Oral & Maxillofacial Pathology

I confirm that I have read this submission and believe that I have an appropriate level of expertise to confirm that it is of an acceptable scientific standard.

## References

[1] : Ectopic upper third molar embedded in a dentigerous cyst of the maxillary sinus: a case report and literature review. *The Egyptian Journal of Otolaryngology* .2024;40(1) : 10.1186/s43163-024-00564-x 10.1186/s43163-024-00564-x

[2] : Diagnosis and Surgical Management of Nasopalatine Duct Cysts. *Journal of Craniofacial Surgery* .2012;23(5) : 10.1097/SCS.0b013e318258764b e472-e474 10.1097/SCS.0b013e318258764b 22976713

[3] : Ectopic Upper Third Molar in Maxillary Sinus: A Case Report and Literature Review. *Indian Journal of Otolaryngology and Head & Neck Surgery* .2022;74(S3) : 10.1007/s12070-021-03039-0 4718-4721 10.1007/s12070-021-03039-0 36742931 PMC9895750

# Reviewer report: Reviewer response for version 2

**DOI:** 10.5256/f1000research.178206.r367849

**Published:** 2025-02-27

**Authors:** Jie Jia

**Affiliations:** 1 Henan University, Kaifeng, Henan, China

1. The revised manuscript is readability. 

 2. The prevalence of MIH both globally and locally is precise.

 3. The explaining about hypersensitivity and restoration style of the affected molar is acceptable.

 Overall, the revised revision is basically meet the requirements, I recommend accept it.

Are enough details provided of any physical examination and diagnostic tests, treatment given and outcomes?

Yes

Is the case presented with sufficient detail to be useful for other practitioners?

Yes

Is sufficient discussion included of the importance of the findings and their relevance to future understanding of disease processes, diagnosis or treatment?

Yes

Is the background of the case’s history and progression described in sufficient detail?

Yes

Reviewer Expertise:

dentist

I confirm that I have read this submission and believe that I have an appropriate level of expertise to confirm that it is of an acceptable scientific standard.

# Reviewer report: Reviewer response for version 1

**DOI:** 10.5256/f1000research.169724.r328480

**Published:** 2024-12-30

**Authors:** Jie Jia

**Affiliations:** 1 Henan University, Kaifeng, Henan, China

The writing of this manuscript is acceptable.

 The exact clinical diagnosis of this patient was correct, and the surgical intervention and orthodontic treatment, as well as restoration of anterior teeth were satisfactory.

 This report presents a comprehensive treatment of odontoma and molar-incisor hypomineralisation in orthodontics. In general, the exact clinical diagnosis of this patient was correct, and the surgical intervention and orthodontic treatment, as well as restoration of anterior teeth were satisfactory. The total  treatment obtained a relatively satisfied effects of this case. I have the following comments for further improvement:

 1. The language of this manuscript need improved.

 2. In the second paragraph of introduction "Moreover, MIH is a prevalent dental developmental disorder that affects approximately 10% to 20% of children and adolescents", the prevalence of  MIH is 2.4%- 40.2% in global, please find the correct reference.

 3. In the Clinical examination part, most index teeth have the defect of MIH. We know that hypersensitivity is the most symptom of MIH. Considering that this patient need to received orthodontic treatment, bleaching, resin infiltration, but the author did not illustrate how to avoid the hypersensitivity during the treatment.

 4. For the restoration of posterior teeth, why perform an indirect onlay restoration for the posterior teeth other than regular resin restoration, and whether show the intraoral effect of the onlay restoration?

 Report presents the successful orthodontic management of a case of odontoma associated with impaction of the mandibular right permanent canine and Molar-incisor-hypomineralization (MIH)

Are enough details provided of any physical examination and diagnostic tests, treatment given and outcomes?

Yes

Is the case presented with sufficient detail to be useful for other practitioners?

Yes

Is sufficient discussion included of the importance of the findings and their relevance to future understanding of disease processes, diagnosis or treatment?

Yes

Is the background of the case’s history and progression described in sufficient detail?

Yes

Reviewer Expertise:

dentist

I confirm that I have read this submission and believe that I have an appropriate level of expertise to confirm that it is of an acceptable scientific standard, however I have significant reservations, as outlined above.

# Response

**Published:** 2025-02-12

**Authors:** Khalid Albawardi

**Affiliations:** Orthodontics, King Abdulaziz Medical City, Riyadh, Riyadh Province, Saudi Arabia

Dear Reviewer,

 Thank you for your thorough evaluation of our manuscript. We appreciate the time and effort you invested in reviewing our work and providing valuable suggestions. Below are our point-by-point responses to your comments:

**1. Language Improvement**

**Comment:**

 “The language of this manuscript needs improvement.”

**Response:**

 We have carefully revised the manuscript to enhance its clarity, grammar, and overall readability. We used professional language editing tools and sought feedback from colleagues to ensure the text is concise, coherent, and consistent throughout.

**2. Prevalence of MIH**

**Comment:**

 “In the second paragraph of the Introduction, the statement ‘Moreover, MIH is a prevalent dental developmental disorder that affects approximately 10% to 20% of children and adolescents’ should be updated. The global prevalence of MIH ranges from 2.4% to 40.2%, with an overall prevalence around 14.3%. Please include correct references.”

**Response:**

 We have revised the second paragraph of the Introduction to more accurately reflect the global prevalence of MIH. In particular, we emphasize that prevalence estimates vary considerably, from 2.4% up to 40.2%, shaped by genetic, environmental, and regional factors. Key references include:

- **Taylor, G. (2017). *Evidence-Based Dentistry***, reporting a prevalence range of 2.4%–40.2%.
- **Zameer et al. (2024). *The Saudi Dental Journal***, indicating that the prevalence of MIH in Saudi Arabia ranges from 8.6% to 40.7%.

Moreover, we have clarified that MIH prevalence in Saudi Arabia ranges from 8.6% to 40.7% (Al-Hammad et al., 2018; Alhowaish et al., 2021). These revisions provide a more comprehensive view of MIH prevalence both globally and locally. The updated content appears in the second paragraph of **Section 1 (Introduction)**.

**3. Clinical Considerations and Hypersensitivity Management**

**Comment:**

 “In the Clinical Examination section, most index teeth show MIH defects. Given that hypersensitivity is a prominent symptom of MIH, how did you address or prevent hypersensitivity during orthodontic treatment, bleaching, and resin infiltration? The manuscript states, ‘According to the MIH-Treatment Need Index (MIH-TNI) criteria, the case was classified as MIH without hypersensitivity and without defect extension (MIH TNI 1).’ However, please discuss strategies to avoid hypersensitivity during these procedures.”

**Response:**

 We appreciate your concern regarding hypersensitivity management. While our case was classified as MIH TNI 1—indicating no evident hypersensitivity or large defect extension at the time of treatment—we have now included a dedicated section detailing strategies to prevent potential hypersensitivity during bleaching, resin infiltration, and orthodontic treatment. These details underscore that, even in TNI 1 cases, proactive measures are prudent to minimize the risk of hypersensitivity. This section was added in **5. Restorative Interventions, Section 5.1: Management of MIH**, specifically in the second paragraph.

**Bleaching**

- Utilize lower-concentration bleaching agents.
- Apply desensitizing agents (e.g., fluoride, calcium phosphate) before and after bleaching.
- Limit contact time of bleaching gels.

**Resin Infiltration**

- Use fluoride varnishes or hydroxyethyl methacrylate-based desensitizers (e.g., Gluma) to occlude dentinal tubules before treatment.
- Apply fluoride or calcium phosphate post-operatively to reinforce enamel and minimize sensitivity.

**Orthodontic Treatment**

- Recommend regular use of desensitizing toothpaste.
- Employ gentle orthodontic forces.
- Consider periodic fluoride varnish or sealant application, especially on affected first molars.

*Reference: Inchingolo AM, Inchingolo AD, Viapiano F, Ciocia AM, Ferrara I, Netti A, Dipalma G, Palermo A, Inchingolo F. Treatment Approaches to Molar Incisor Hypomineralization: A Systematic Review. Journal of Clinical Medicine. 2023 Nov 20;12(22):7194.*

**4. Rationale for Indirect Onlay Restoration**

**Comment:**

 “For posterior teeth restoration, why did you choose an indirect onlay restoration instead of a direct resin restoration, and can you provide intraoral images showing the effect of the onlay?”

**Response:**

 We opted for an indirect onlay restoration on the upper right first molar due to extensive cusp involvement and weakened tooth structure that exceeded what a conventional direct composite could reliably support. Indirect onlays provide superior control over occlusal anatomy, marginal adaptation, and material properties (e.g., wear resistance), offering better long-term stability and protection for compromised tooth structure.

 In response to your feedback, we have updated the manuscript with additional details and, where possible, intraoral photographs illustrating the final outcome of the onlay restoration. These visuals support our treatment choice and demonstrate its clinical success. The photo has been added to **Section 5.2.3: Restoration of Posterior Teeth** at the end of the paragraph.

 We sincerely thank you for your invaluable comments. Your insights have significantly helped us improve the clarity, scientific accuracy, and clinical relevance of our manuscript. We trust that our revisions address all your concerns and look forward to any additional feedback you may have.
